# Cilia-independent gas–liquid transport, a third mechanism mediating airway mucus clearance

**DOI:** 10.1172/JCI182984

**Published:** 2026-03-17

**Authors:** Siddharth K. Shenoy, Mark Gutay, Ian Brown, Troy D. Rogers, Kane Banner, Nico Olegario, Nicholas Griffin, Henry P. Goodell, Bryan Yoder, David S. Lalush, David A. Edwards, Richard C. Boucher, Barbara R. Grubb, Brian Button

**Affiliations:** 1 Marsico Lung Institute,; 2 Department of Biomedical Engineering, and; 3 Department of Biochemistry and Biophysics, University of North Carolina, Chapel Hill, North Carolina, USA.; 4 Center for Nanomedicine, Johns Hopkins University Medical School, Baltimore, Maryland, USA

**Keywords:** Cell biology, Pulmonology, Transport

## Abstract

Airway mucus clearance from the lungs occurs by 2 widely recognized mechanisms: cilia-mediated clearance and high-velocity airflow-mediated cough clearance. However, a potentially important third mechanism of mucus clearance, referred to as cilia-independent gas–liquid transport (GLT), was proposed based on in vitro model systems to occur during normal tidal breathing but has largely been overlooked. We conducted in vitro and in vivo studies to investigate the role of tidal breathing airflow rates in mucus clearance. An in vitro airway culture bead-tracking model demonstrated airflow-dependent mucus transport at tidal breathing flow rates. As with other modes of mucus clearance, GLT was critically dependent on mucus concentration. In vivo studies in cilial beat-deficient mice demonstrated that GLT-mediated mucus clearance occurs during tidal breathing in the absence of cough, and the rate of GLT mucus clearance was dependent on breathing frequency and body orientation. These studies demonstrated that GLT is a third mechanism of mucus clearance and likely represents a significant mode of clearance in persons with cilial dysfunction. These findings indicate that increasing breathing rates through exercise, using mucus rehydrating agents or mucolytics, or combining these approaches may restore clinically and physiologically meaningful airway clearance in these patients.

## Abstract 2

Gas–liquid transport-mediated mucus clearance exists as a third mechanism of mucus clearance and is dependent on mucus concentration, breathing rate, and orientation of the body.

## Introduction

The human lung contains the largest epithelial surface area of the body (~100 m^2^) and is continually exposed to inspired air containing pathogens and environmental particulates (1). The health of the conducting airways is maintained by the entrapment of inhaled particulates and pathogens in a thin, protective viscoelastic mucus layer that lines airway surfaces. Mucus is continually transported from the lungs by coordinated ciliary beating (2), a process termed mucociliary clearance (MCC). In addition to MCC, mucus can be cleared by cough clearance (CC), a reflex mechanism that utilizes high-speed airflow (up to 200 m/s) to expel mucus accumulated in central airways from the lungs (3–5). Cough is especially important during allergen exposure or lung infection, conditions that produce concentrated, adherent mucus that is resistant to cilial-dependent clearance (6–9). During cough, the rapid expiratory airflow generates pronounced mucus wave motion along the airway surfaces, producing shear forces that mobilize the mucus layer as a 2-phase gas–liquid flow.

While clearance of mucus by cilia-dependent MCC and high-speed airflow-dependent CC has been well described, a third mechanism of mucus clearance, referred to as cilia-independent gas–liquid transport (GLT), was proposed in the 1980s (10). Like CC, GLT was proposed to reflect a 2-phase gas–liquid flow-dependent mechanism (11, 12) by which gas (air) moves an underlying mucus (gel) layer over airway epithelial surfaces. However, unlike the high-speed flow of air generated during a cough, GLT is predicted to occur at airflow velocities associated with tidal breathing. Kim and colleagues (12) used various model systems to demonstrate that linear airflow velocities of 1–3 meters per second could propel mucus via GLT. Importantly, they predicted that such airflow velocities could be achieved during the expiratory phase of high minute volume (100 L/min) breathing throughout the airways to the level of the bronchioles (12, 13).

**Figure 1:**
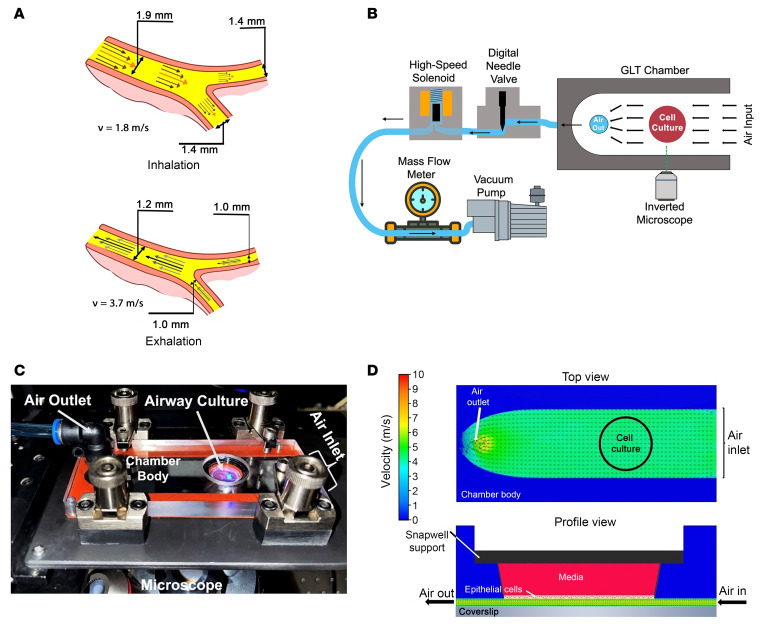
Model to study airflow-mediated mucus clearance. (**A**) Model of a eighth-generation airway to show airflow asymmetry during inhalation (top) and exhalation (bottom) at 75 L/min volumetric flow (exercise). During inhalation, airways dilate to facilitate air entry into the lung. Conversely, during exhalation, airways narrow. If the inspiratory and expiratory volumes are similar, the airflow velocities and shear stresses are higher on exhalation, which promotes the net clearance of mucus from the lungs. (**B**) Diagram of the in vitro GLT system showing the airflow pathway (see Methods for additional details). (**C**) Photo of the system used to deliver breathing-mimicking airflow pulses to human bronchial airway cultures, mounted on an inverted fluorescence microscope. (**D**) Illustrations showing the chamber from top down (top) and profile (bottom) combined with computational fluid dynamic simulation of the laminar airflow during a 5 m/s airflow pulse.

A requisite for GLT-mediated mucus clearance from the lungs is that the expiratory airflow velocity exceeds inspiratory airflow velocity (12). While the inspired and expired air volumes per unit time are similar during tidal breathing, the airways exhibit a reduction in diameter during exhalation compared with inhalation (13, 14). This hysteresis in airway diameter during tidal breathing reflects the effects of intrathoracic pressures during the breathing cycle. Pedley et al. (13) quantified airway diameters during tidal breathing, demonstrating that intrathoracic pressure changes alter the airway caliber by 5%–25%, depending on the airway generation. This hysteresis increases expiratory relative to inspiratory airflow rates, an asymmetry hypothesized to produce net mucus transport in the cephalad direction (10, 12, 15, 16) (Figure 1A). The effectiveness of GLT-mediated mucus clearance, however, is influenced by several airway and breathing dynamics factors, including airway geometry and compliance, airflow velocity, breathing frequency, mucus concentration with its associated effects on mucus viscoelastic properties, and mucus–epithelial surface adhesion (12, 16, 17).

GLT-mediated mucus clearance is potentially important in individuals who exhibit defects in ciliary motility (18), including primary cilia dyskinesia (PCD), and in airway regions devoid of functional cilia consequent to airway damage, e.g., due to gastric aspiration (19, 20), viral infection (21), or cigarette smoking (22). However, functional studies investigating the significance of GLT, as well as biophysical studies defining the variables that regulate its efficiency in health or disease, are lacking. To address this gap, we developed in vitro models to study mucus flow at airflow velocities spanning normal tidal breathing to those evident during exercise. In parallel, we performed in vivo studies to evaluate the role of GLT-mediated transport in WT mice, mice with defective ciliated cell numbers or cilia beating (PCD), and neonatal mice and neonatal ferrets exhibiting reduced or absent ciliation at birth (23, 24).

## Results

### In vitro model system to measure GLT.

An airflow–fluid dynamics model was generated to describe airflow rate hysteresis at both tidal breathing (15 breaths/min at 15 L/min) and moderate exercise (50 breaths/min at 75 L/min) (Figure 1A and Table 1) based on Weibel’s anatomical model of the human respiratory system (25, 26) and Pedley and Schröter’s measurements of airway diameter changes during inhalation and exhalation (13). Under tidal breathing conditions, airflow pulses remained laminar (Reynolds numbers < 2,000; Table 1), whereas flow rates in larger airway generations (1–4) during exercise are predicted to be transitional or turbulent. To investigate airflow–mucus transport dynamics experimentally, we developed a system that delivered unidirectional pulses of humidified air to airway epithelial cultures with a well-characterized endogenous mucus layer (27), mounted on an inverted fluorescence microscope interfaced to a high-speed camera (Figure 1, B–D). This setup enabled precise quantification of the relationships between airflow velocity, pulse duration, mucus concentration, and mucus translocation (28, 29). For these studies, we selected a pulse velocity of 1 m/s, which permitted accurate measurements of mucus translocation while mimicking exhalation airflow velocities in airway generations 0–6 (Table 1). A higher velocity of 5 m/s was chosen to replicate exercise-induced flows.

### Evidence of breathing-relevant GLT in vitro.

**Figure 2:**
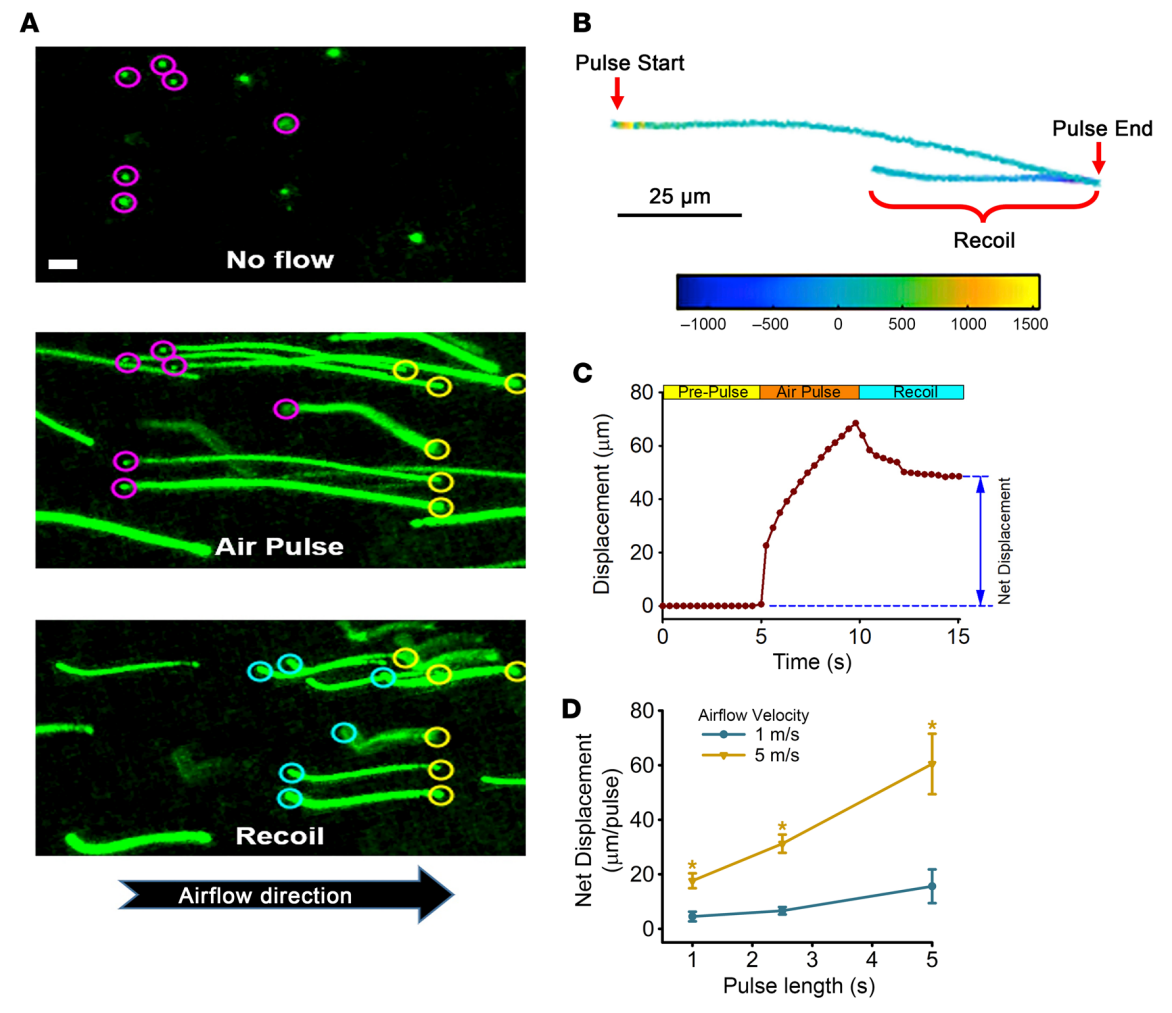
Airflow-mediated mucus transport across HBE cells. (**A**) Representative 5 s time-lapse microscopy images of fluorescent particles before (top), during (middle), or after (bottom) a 5 m/s airflow pulse. The starting points are indicated by magenta circles, the positions at the end of the air pulse by yellow circles, and the positions at the end of the elastic recoil by cyan circles. Scale bar: 10 μm. (**B**) Mucus transport rates were evaluated by tracking fluorescent tracers during the time-lapse video. This representative particle trace shows displacement before, during, and after a 5 s airflow pulse in normal (10 mg/mL) mucus. The gradient scale represents discrete, instantaneous, point-to-point velocity in μm/s. (**C**) Displacement plot (μm) versus time (seconds) for normal 10 mg/mL mucus in response to a 5 s airflow pulse at 5 m/s. The net displacement was evaluated at the end of the recoil period. (**D**) Comparison of net displacement for various air pulse lengths (from 1 to 5 s) at 2 airflow velocities (1 or 5 m/s). *P < 0.01 versus 1 m/s at each pulse length; 1-way ANOVA followed by Tukey’s post hoc test.

Initial studies were performed in human airway bronchial epithelial (HBE) cultures covered with mucus with a concentration consistent with health (10 mg/mL organic content; ~1% organic solids) (30), into which 1 μm fluorescent particles were incorporated to aid tracking of mucus flow by high-speed microscopy (31) (Figure 1B). Mucus flow across the airway surface was recorded while subjected to an airflow pulse protocol approximating tidal-breathing parameters over a period of 15 s (5 s of no flow, 5 s of air pulse, and 5 s of elastic recoil). Representative time-lapse traces in Figure 2A show that (a) during airflow velocities simulating a peak tidal expiratory flow rate of 5 m/s, particles embedded in a normally concentrated (10 mg/mL solids) mucus layer exhibited flow in the direction of airflow during the pulse (Air Pulse), and (b) upon cessation of airflow, a retrograde movement of mucus in the opposite direction (Recoil) was detected, reflecting the elastic element of a mucin-containing mucus layer (30). Point-to-point tracking of fluorescent particles embedded in the mucus layer was used to monitor mucus flow throughout the pulse protocol (7) (Figure 2B). During the active airflow pulse, mucus displacement was biphasic: an initial rapid velocity followed by a slower, steady-state velocity for the remainder of the pulse duration (Figure 2C). The imaging data were used to calculate ensemble mucus (bead) displacements relative to the starting position (no airflow) during the air pulse (Figure 2, C and D). At the end of the air pulse, the elastic recoil of the viscoelastic mucus layer resulted in a retrograde flow of mucus. The difference between the starting and postrecoil positions was defined as the net displacement of a breath-mimicking unidirectional airflow pulse.

To investigate the dependence of air pulse velocity and duration on the net displacement of mucus during simulated exhalation at rest versus exercise, studies were performed over a range of pulse lengths (from 1 to 5 s) at either 1 or 5 m/s, respectively (Figure 2D). Mucus displacement during the breath-mimicking air pulse increased directly with the pulse length, irrespective of airflow velocity. In contrast, net mucus displacement did not scale proportionally to airflow velocity. At 5 m/s, net mucus displacement was 7.5 ± 2.0 fold higher than values obtained at 1 m/s airflow, consistent with mucus shear-thinning behavior (elasticity decreases with increasing stress) at higher airflow velocities.

### Mucus hyperconcentration reduces GLT-mediated clearance.

**Figure 3:**
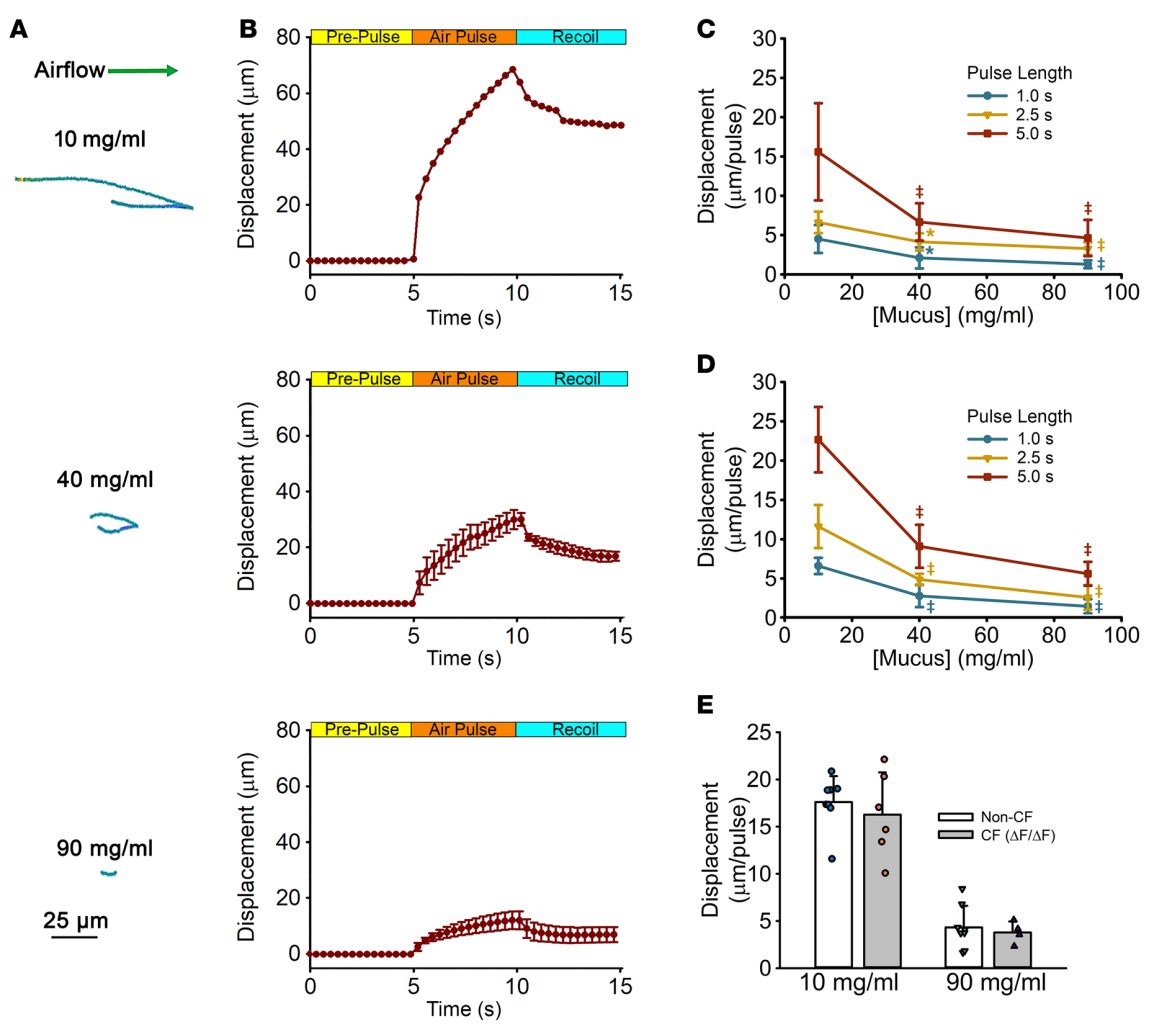
Concentration dependence of airflow-mediated mucus transport. (**A** and **B**) Representative bead tracings as a function of mucus concentration (**A**) and summary net displacement plots (**B**) in response to a 5 s airflow pulse at 5 m/s in the presence of healthy (10 mg/mL; top), mild disease (40 mg/mL; middle), or severe disease (90 mg/mL; bottom) mucus. Scale bar: 25 μm. (**C** and **D**) Effect of mucus concentration and pulse length at 1 m/s (**C**) and 5 m/s (**D**) airflow on net mucus displacement (μm/pulse). *P < 0.05, ^‡^*P* < 0.01, 1-way ANOVA followed by Tukey’s post hoc test. (**E**) Comparison of GLT rates between mucus produced from nondiseased (non-CF) and CF cultures at 1 and 5 m/s (not significant, unpaired, 2-tailed Student’s *t* test).

Most, if not all, muco-obstructive airway diseases, including chronic bronchitis, asthma, cystic fibrosis (CF), and PCD, are characterized by hyperconcentrated mucus (32). To investigate the effect of mucus concentration on the efficiency of GLT-mediated mucus transport, in vitro studies were performed over a range of mucus concentrations spanning normal (10 mg/mL; 2% total solids); moderately dehydrated (hyperconcentrated) mucus (40 mg/mL; 5% total solids) representing PCD (33–35) and chronic bronchitis (7); and severely dehydrated (hyperconcentrated) mucus (90 mg/mL; 10% total solids), mimicking the concentration of mucus removed from CF airways after lung excision (31). Similar to previous studies on the clearance of mucus by cilia and cough (7, 30, 36), GLT-mediated mucus flow was significantly affected by mucus concentration. Figure 3, A and B, show representative mucus tracks and displacement plots of 5 m/s air pulses (for 5 s) across HBE cultures covered by a normal (10 mg/mL), mildly dehydrated (40 mg/mL), or severely dehydrated (90 mg/mL) mucus layer. As the mucus concentration increased, a significant decrease in the net transport of mucus was observed at both 1 m/s (Figure 3C) and 5 m/s (Figure 3D) airflow velocities. Notably, GLT was negligible at mucus concentrations representing severe disease-like conditions (e.g., 90 mg/mL mucus). However, in cases of mild dehydration (33), e.g., 40 mg/mL mucus, GLT rates increased with increasing airflow velocities from 1 to 5 m/s.

Because these studies were performed in normal airway cultures with hyperconcentrated normal airway mucus, we performed similar studies with cultures obtained from CF donor lungs. Both CF and non-CF cultures exhibited GLT clearance rates that were comparable at mucus concentrations of 10 mg/mL (Figure 3E). Similarly, GLT on both CF and non-CF cultures was significantly reduced when mucus concentrations were increased to values associated with endogenous CF values (Figure 3E).

### Improvement of GLT efficiency with muco-active agents.

**Figure 4:**
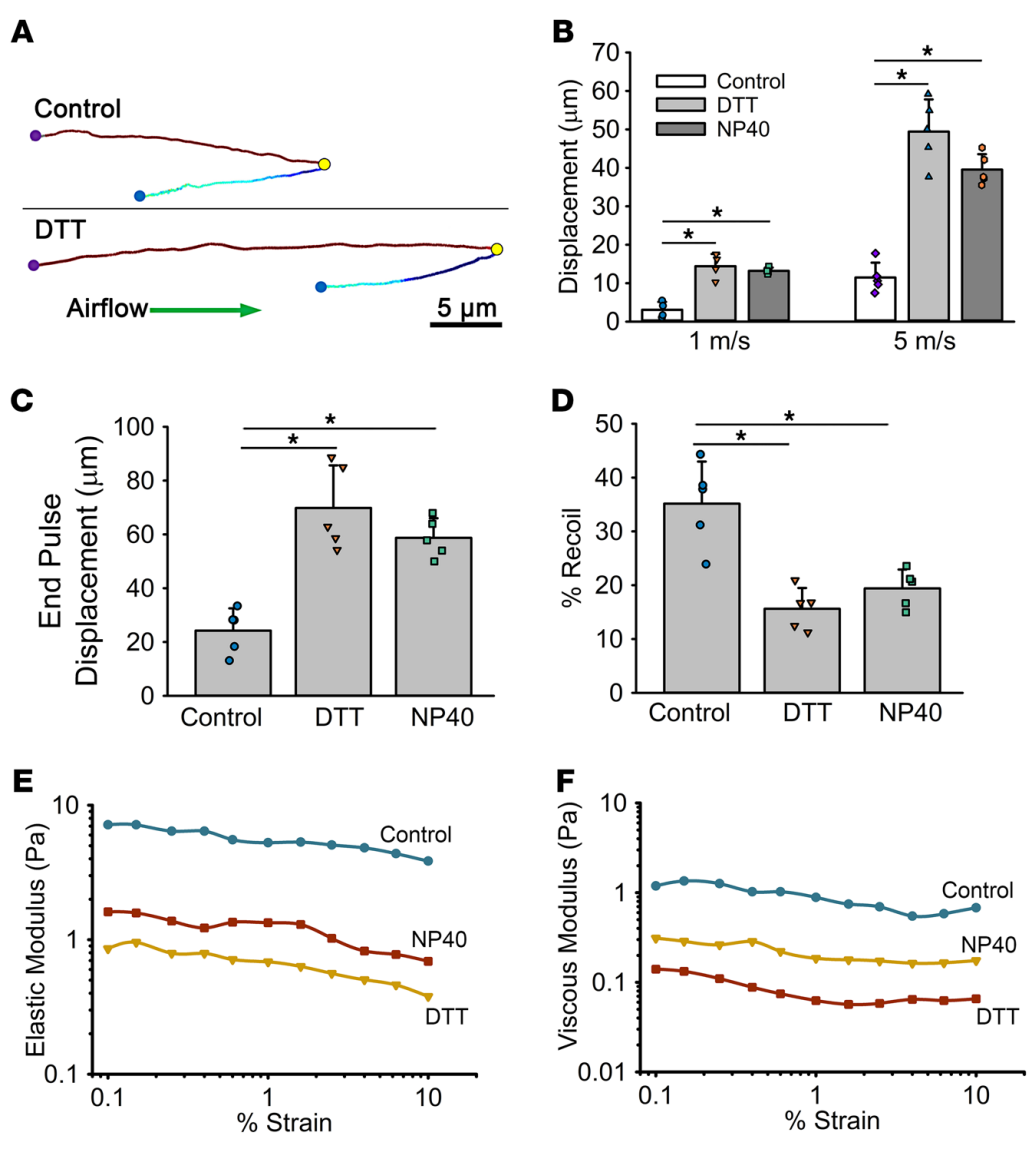
Therapeutic treatment to improve GLT-mediated transport. (**A**) Representative bead traces in control mucus (40 mg/mL) or 30 min after DTT (10 mM) addition. Purple, bead start location; yellow, bead position at the end of the air pulse (5 m/s for 2.5 s); blue, bead position at the end of the recovery period. Scale bar: 5 μm. (**B**) Summary of net displacement of mucus (40 mg/mL) under control conditions (0.9% PBS) and 30 min after the nebulization of a reducing agent (DTT; final concentration 10 mM) or surfactant (NP-40; 0.01%) at either 1 or 5 m/s for 2.5 s. (**C**) Comparison of end pulse distances (i.e., the distance between the purple and yellow circles in **B**) for a 2.5 s pulse at 5 m/s. *P < 0.01, 1-way ANOVA followed by Tukey’s post hoc test. (**D**) Comparison of the magnitude of elastic recoil (measured as the percent recovery after the termination of the airflow pulse, i.e., distance between yellow and blue circles in **B**). *P < 0.01 between groups. (**E** and **F**) Effect of DTT (10 mM) and NP-40 (0.01%) on the elastic (**E**) and viscous (**F**) modulus of a 40 mg/mL mucus layer over a range of oscillatory strains (at 6.25 rad/s) as measured with a cone-and-plate rheometer.

The finding that higher mucus concentrations reduce airflow’s effectiveness in clearing mucus suggests that therapies that reduce mucus concentration or alter mucus properties by other mechanisms may improve GLT-mediated clearance. Such therapies might also target the mucus frictional and adhesive interactions between the secreted and tethered mucins that influence mucus mobility. To test the notion that muco-active agents may be effective in muco-obstructive diseases with moderate mucus dehydration, studies were performed by nebulizing small volumes (100 nL/cm^2^) (37)onto mucus (40 mg/mL) containing either (a) a reducing agent (10 mM DTT) to decrease mucin polymer length/molecular weight (38) or (b) a surfactant (0.01% NP-40 [IGEPAL CA-630]) to decrease mucus–cell surface interactions (36, 38). The increase in net mucus displacement after DTT treatment versus control (40 mg/mL mucus) was observed in representative bead tracings in response to a 5 m/s pulse (for 2.5 s) (Figure 4A). Cumulative data from the muco-active agent administration protocol revealed that both DTT and NP-40 increased net bead displacement at 1 and 5 m/s pulses (Figure 4B). Both agents increased particle displacement during the pulse (Figure 4C) and reduced the recoil distance of the mucus (Figure 4D). These data are consistent with reductions in both the elastic (Figure 4E) and viscous (Figure 4F) moduli of mucus, as measured by traditional cone-and-plate rheology, and a reduction in the viscous dissipation at the mucus–cell surface interface (36). Additionally, because we have shown that these agents affect the adhesive interactions with the cell surface, it is possible that a reduction in mucus adhesive strength also contributed to accelerated clearance by GLT (36). Collectively, these data suggest that muco-active agents targeting both intrinsic mucus properties and mucus interactions with the cell surface may enhance GLT-mediated clearance of moderately dehydrated mucus.

### In vivo evidence of GLT to clear mucus in the absence of cilia and cough.

**Figure 5:**
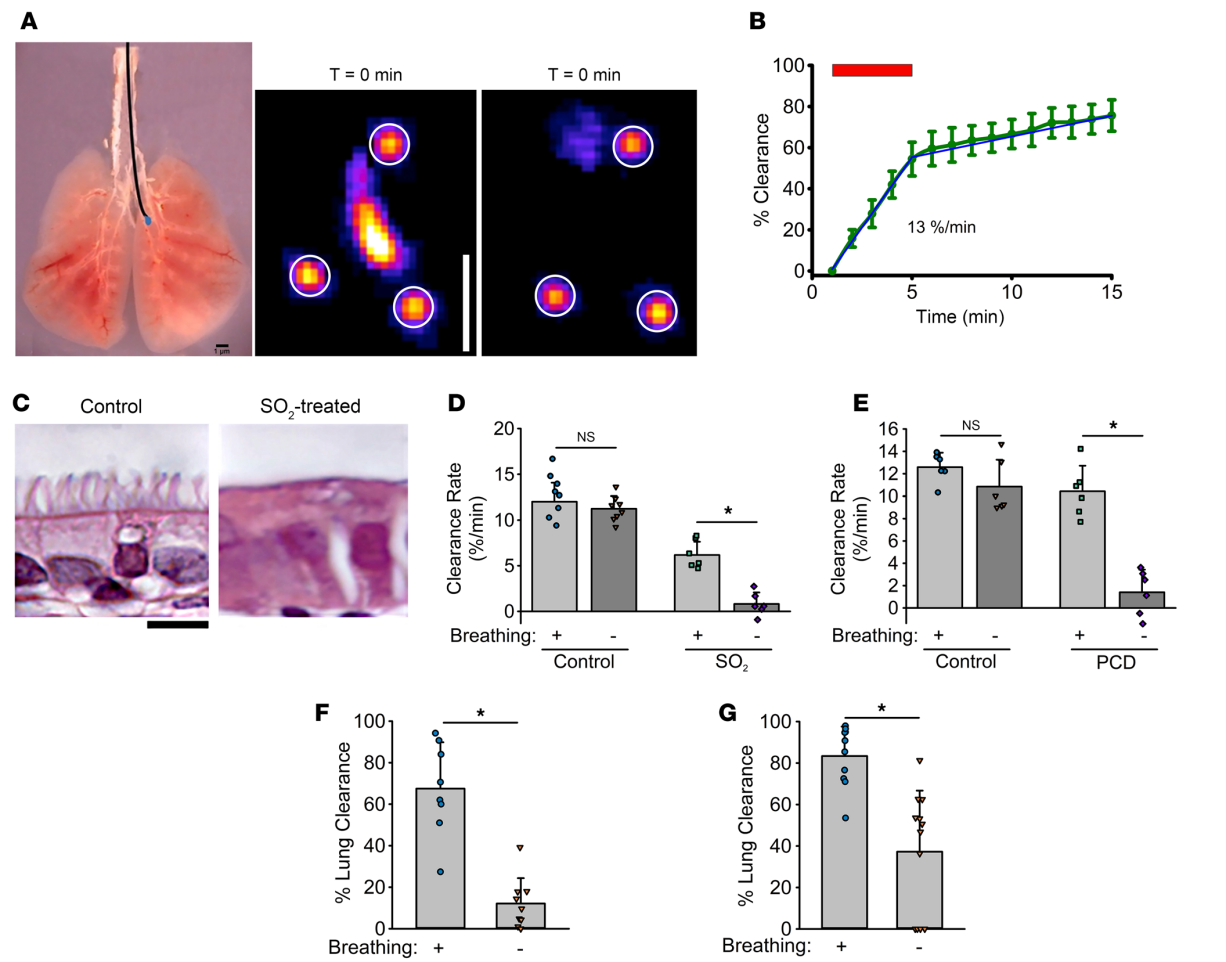
GLT-mediated mucus clearance in mice and ferrets. (**A**) Left: Technical representation of depositing a small amount (~150 nL) of ^9139m^TC-SC radiotracer (blue drop) in the proximal airways of mice using a narrow (0.6 mm OD) polyethylene tube (black). Scale bar = 1 μm. Right: Representative γ camera images showing the clearance of ^99m^TC-SC after 5 min. Three subdermally implanted fiducial ^99m^TC markers (circled) were used for image alignment. Scale bar = 1 cm. (**B**) Representative plot showing the mucus clearance versus time in a normal mouse, determined from time-lapse γ camera images as the percentage of ^99m^TC-labeled particles cleared from the trachea over 15 min. The initial clearance rate was calculated over 0–5 min (red bar). (**C**) Histological cross sections (H&E staining) showing the ablation of cilia from the lumen of SO_2_-treated animals (right) compared with controls (left). Scale bar: 10 μm. (**D**) Summary of the peak clearance rates (first 5 min) from sham-treated control mice versus mice with cilia chemically ablated by SO_2_ exposure. Mucus clearance rates are shown from alive (breathing +) versus dead (breathing –) control and SO_2_-treated animals. (**E**) Summary of mucus clearance rates from PCD mice with defective cilia. Littermates with functional cilia served as genetic background controls. (**F**) Comparison of total lung clearance in neonatal (P1–P2) mice with poor ciliation, 15 min after installation of 3 μm fluorescent particles in breathing versus nonbreathing (i.e., after euthanasia) animals. (**G**) Total lung clearance in breathing versus nonbreathing neonatal ferrets (P1–P2). *P < 0.01, unpaired 2-tailed Student’s *t* test.

The in vitro studies demonstrated that mucus is transported across the airway surface by airflow at velocities simulating tidal breathing. However, our in vitro model only assessed mucus transport in response to unidirectional airflows calculated to mimic human in vivo conditions. To assess whether asymmetric airflow associated with tidal breathing produces net mucus clearance in vivo, GLT studies were performed in 4 animal models with or without functional cilia. Mucus transport was measured as the clearance rate of a small volume (150 nL) of radiolabeled sulfur colloid particles (^99m^Tc-SC) (8), focally deposited in the left murine mainstem bronchus (Figure 5A). The clearance was measured by γ scintigraphy, and rates were calculated as a percentage of particles cleared from the airways over time (39) (Figure 5A). Similar to radiotracer clearance studies in humans (9), ^99m^Tc-SC clearance from mice exhibited biphasic kinetics (Figure 5B) with a rapid clearance of the majority of particles followed by a slower, more continuous, rate of clearance.

As a first test of GLT-mediated clearance in vivo, control mice were compared with a mouse model in which ciliated cells were ablated chemically by inhalation of sulfur dioxide (SO_2_) gas (500 ppm for 3 hours). SO_2_ exposure was used previously to produce acute airway injury (40, 41) and shedding of luminal airway epithelial cells (including ciliated cells), leaving only an intact basal cell layer for up to 7 days before cilia reappear (42). Histologic sections of mouse bronchial specimens harvested 3 days after SO_2_ treatment confirmed that SO_2_ inhalation produced an airway surface devoid of ciliated cells compared with control animals (Figure 5C). Three days after exposure to SO_2_ (or sham control), the clearance of mucus by cilia beating versus tidal breathing (GLT) was studied under 2 conditions: (a) alive, anesthetized, and spontaneous breathing and (b) immediately after death (i.e., nonbreathing). Notably, ciliary beating and MCC remain active for at least 3 hours postmortem in control mice (43). Consistent with this observation, mucus clearance rates were similar between breathing and nonbreathing conditions in normal control mice (Figure 5D). Living and breathing SO_2_-treated mice cleared the deposited tracer in the absence of cilia at rates approximately 50% of those exhibited by sham-exposed living control mice (5.8% ± 1.6%/min vs. 11.1% ± 1.2%/min) (Figure 5D). Importantly, neither coughing nor labored breathing was detected in either control or SO_2_-treated animals during the γ imaging studies. After death and cessation of breathing, the SO_2_-treated mice exhibited no measurable particle/mucus clearance, demonstrating the role of GLT in mediating mucus clearance during tidal breathing conditions.

The role of GLT in airway mucus clearance was also tested in a mouse model with genetically dysfunctional cilia. This mouse model of PCD manifests a deletion in the intermediate dynein chain (Dnaic1) and has previously been reported to exhibit deficient MCC (44). To investigate whether PCD mice exhibit GLT, ^99m^Tc-SC radiotracer clearance was measured using protocols identical to those for the SO_2_-treated mice. These results demonstrated that in live breathing PCD mice, mucus was cleared at nearly control (non-PCD) rates (8.3% ± 1.4%/min). As with SO_2_-exposed mice, mucus clearance was abolished in nonbreathing, acutely euthanized PCD mice, whereas non-PCD littermate controls cleared mucus effectively immediately after euthanasia (8.3% ± 0.9%/min) (Figure 5E).

As final tests of the role of GLT in vivo, both neonatal mice and ferrets were studied. At birth, airways in both species are poorly ciliated, with cilia covering approximately 20% of the airway surfaces (23, 31). Ciliation increases through P14, but before approximately P9, cilia exhibit poor beat coordination, which, combined with reduced cilial cell numbers, produces virtually no measurable cilia-mediated flow across the airway epithelial surface in either species (23, 31). Previous studies of neonatal animal mucus clearance rates utilized a fluorescent bead technique to measure clearance in alive/breathing versus dead/nonbreathing conditions (45). Using this approach, clearance in breathing (alive) neonatal mice was observed to be significantly greater than clearance in euthanized, nonbreathing conditions (Figure 5F). Similar findings were observed in neonatal ferrets (P1–P2) (Figure 5G). Collectively, these data demonstrate that tidal breathing–mediated GLT provides significant clearance of mucus when cilial-dependent clearance is ineffective in the neonatal period.

### Breathing frequency increases GLT rate.

**Figure 6:**
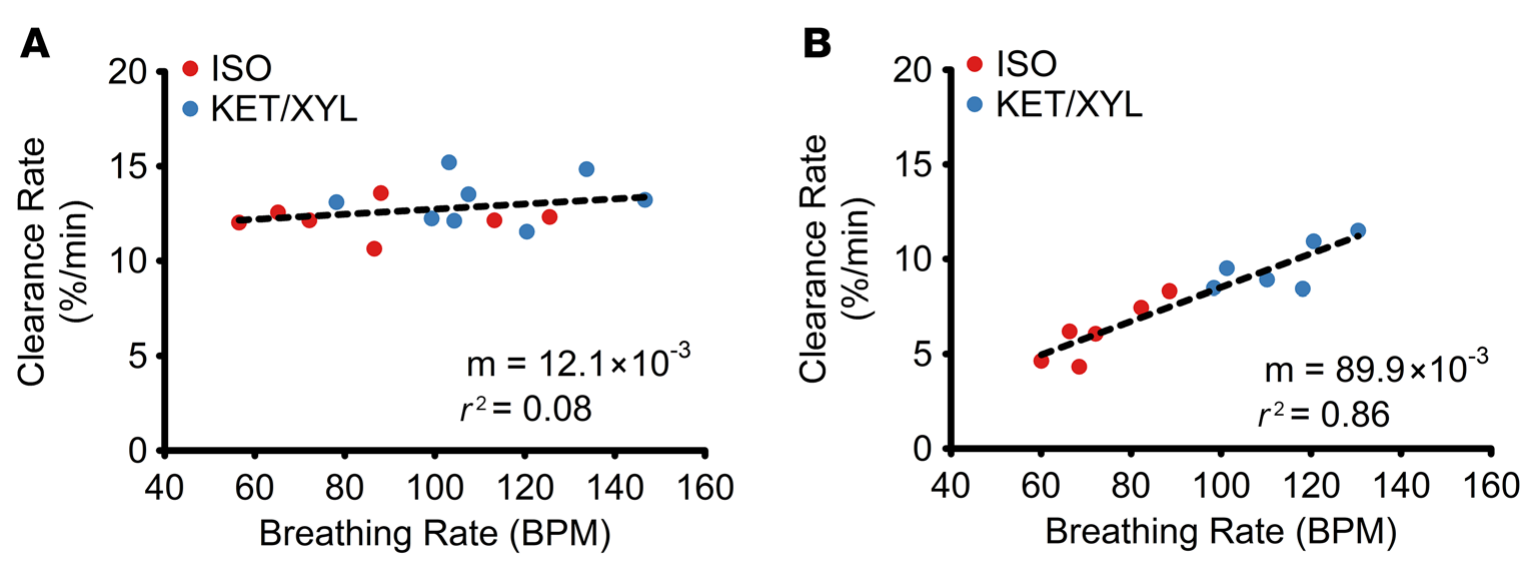
Effects of breathing rate on cilia- versus airflow-mediated mucus clearance in vivo. ^99m^TC-SC clearance rates versus breathing frequency (in bpm) in spontaneously breathing control (**A**) and SO_2_-treated (**B**) mice. Data are shown from individual isoflurane (ISO) anesthetized animals (red) and ketamine/xylazine anesthetized (KET/XYL) animals (blue). Slope (m) is shown as % clearance/min/bpm. In both sham control and SO_2_-treated mice, isoflurane-breathing mice exhibited significantly reduced breathing rates compared with ketamine/xylazine anesthetized mice: 85.9 ± 24.4 versus 111.7 ± 21.4 bpm in control mice (*P* < 0.05) and 73.1 ± 10.9 versus 115 ± 11.7 bpm (*P* < 0.01) in SO_2_-treated mice.

Higher breathing frequencies (i.e., higher number of GLT-mediated pulses/min) are predicted to produce greater net GLT-mediated mucus clearance per min. Accordingly, the impact of breathing frequency on the rate of GLT in vivo was investigated. In these studies, the rates of mucus clearance versus breathing rate frequency were measured in anesthetized control and SO_2_-exposed mice. Breathing frequency in spontaneously breathing mice was varied using 2 anesthetic regimens: (a) ketamine/xylazine, which has mild effects on breathing rate (46); and (b) isoflurane, which produces significantly slower breathing rates (47). A summary of GLT-mediated clearance versus breathing rate in control and SO_2_-treated animals is shown in Figure 6. In control animals with functional cilia beating, there was no correlation between breathing frequency and clearance (*R*^2^ = 0.08) (Figure 6A). In contrast, a strong correlation was observed (*R*^2^ = 0.86) between breathing frequency and the rate of GLT-mediated mucus clearance in SO_2_-treated animals (Figure 6B). While these studies were performed with different general anesthetics to achieve a range of breathing frequencies, the dependence of clearance rate on breathing frequency in SO_2_-treated mice was similar for the breathing rates for both anesthetics. Together, these data suggest that breathing frequencies govern the rate of GLT-mediated mucus clearance when cilia beating is defective.

### GLT is influenced by gravitational forces.

**Figure 7:**
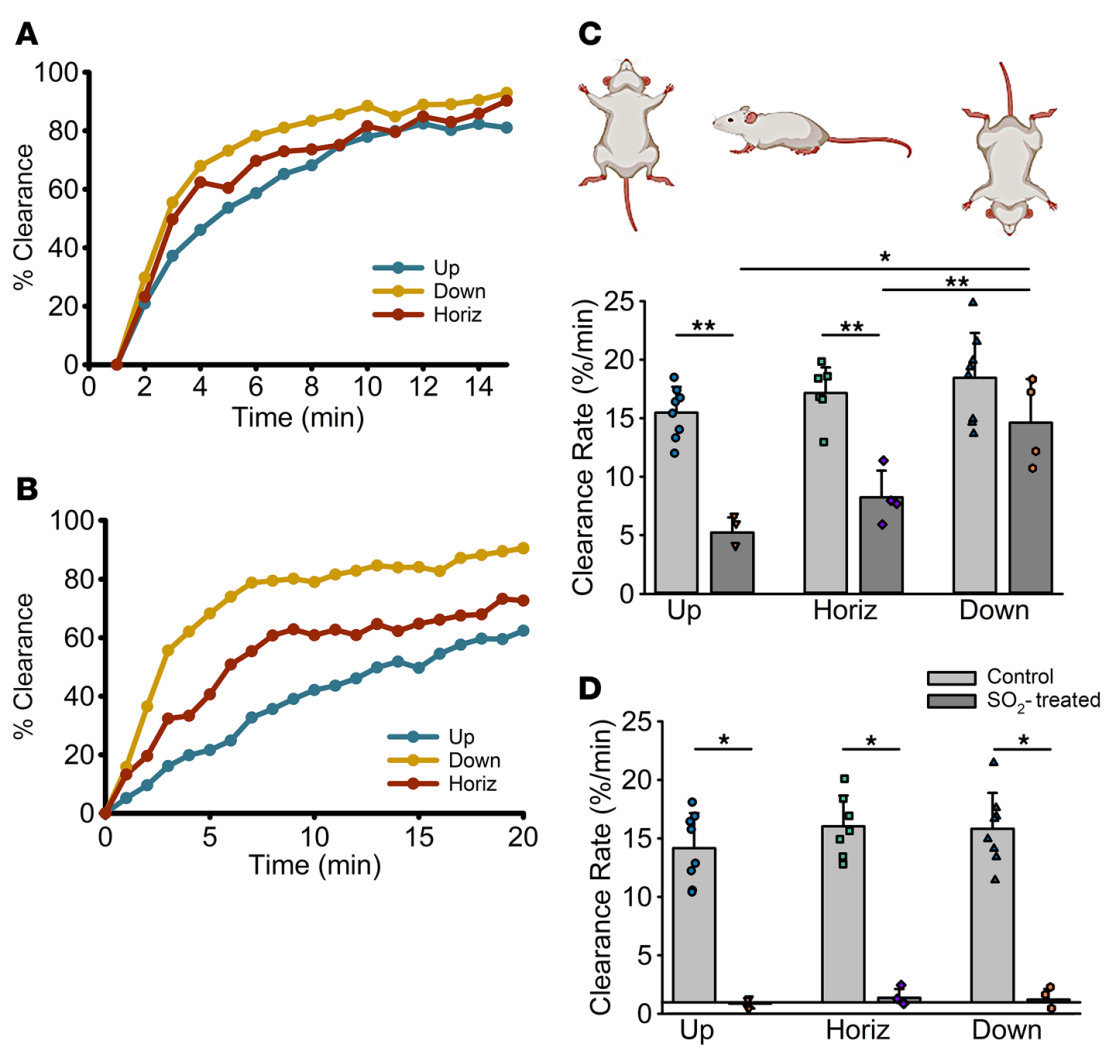
Effects of body orientation on airflow-mediated mucus transport. (**A** and **B**) Comparison of ^99m^TC radiotracer clearance in sham-treated control (**A**) and SO_2_-treated (**B**) animals when positioned horizontally (Horiz) versus a heads-up or heads-down orientation. (**C** and **D**) Summary of the mucus clearance rates in % clearance/minute (for the first 5 min) in alive/breathing (**C**) and dead/nonbreathing (**D**) mice. *P < 0.01, **P < 0.05, 1-way ANOVA followed by Tukey’s post hoc test.

We further evaluated how changes in body position, which alter gravitational forces (48, 49), impact cilia-dependent and GLT-mediated mucus clearance in vivo. To study this variable, mucus clearance studies were performed with sham-treated control and SO_2_-treated mice positioned (a) horizontally (neutral to gravity), (b) heads-up/vertically (against gravity), and (c) heads-down (with gravity). Mucus clearance was measured in the trachea, which has the advantage of having a single orientation that follows body position. In living, breathing control mice, body position had little effect on the rate of clearance (Figure 7, A and C), with the heads-down position having only slightly faster clearance rates (18.5% ± 1.36%/min), not significantly different from horizontal (16.0% ± 1.82%/min) or heads-up animals (15.48% ± 2.21%/min). In contrast, in alive breathing mice lacking cilia (SO_2_ treated), the rates of mucus clearance were significantly affected by body orientation, with clearance significantly faster with (heads-down) versus against (heads-up) gravity (Figure 7, B and C). In the nonbreathing (posteuthanasia) animals, body position had minimal impact on mucus clearance in control mice, and, as predicted in dead SO_2_-exposed animals, all mucus transport ceased regardless of body position (Figure 7D).

## Discussion

Mechanical clearance of mucus from the lung by cilia beating constitutes the airway’s primary defense mechanism, and the failure of this system contributes to the pathophysiology of muco-obstructive lung diseases (30, 32). In conditions in which cilia-mediated clearance is insufficient, cough serves as a backup mechanism. However, a third mucus clearance mechanism, i.e., GLT, was proposed over 4 decades ago based on biophysical models that suggested airflow-mediated mucus clearance occurred at airflow velocities associated with normal tidal breathing (12).

Kim and colleagues (10) proposed GLT based on investigations of the flow of Newtonian fluids in rigid tubes subjected to tidal breath–mimicking airflow rates. While innovative, these artificial tube in vitro studies lacked key physiological parameters characteristic of airways, including a non-Newtonian viscoelastic fluid (i.e., mucus) and a surface that mimics interactions of mucus with airway surfaces. A subsequent study by Fink (15) provided foundational evidence for 2-phase GLT in vivo, demonstrating that inverse ratio ventilation with inspiratory/expiratory ratios of 1.9:1 and 3:1, which produced high expiratory relative to inspiratory flow rates, effectively cleared 30%–49% of a deposited mucus simulant (polyethylene oxide, viscosity 80 poise, elastic modulus 30 dyne/cm²) from the mainstem bronchi of mechanically ventilated sheep. This study confirmed that when airflow velocities are greater during expiration than inspiration, GLT is generated in vivo. These findings suggested the potential of inverse ratio ventilation to manage excessive bronchial secretions in ventilated patients. However, the study’s reliance on nonphysiologic ventilation ratios and expiratory airflow rates beyond those of physiologic tidal breathing limited the extension of this technique to clinical practice.

To investigate the physiologic relevance of GLT-mediated clearance in humans, we first developed an in vitro model system to administer to human airway epithelial culture surfaces physiologically relevant airflow pulses and measure airflow-mediated transport of endogenous mucus using high-speed video microscopy (30, 25). The airflow rates studied in the in vitro protocols were chosen based on flow rates estimated to be manifest in humans in vivo, as calculated from mathematical models of Poiseuille viscous pressure drop reported by Pedley et al. (13) and Weibel’s anatomical representation of the human respiratory system (Table 1) (26, 50).

Experimentally, airflow rates of 1 m/s were required to make accurate measurements of mucus–bead displacement, suggesting that approximately 1 m/s is the minimum airflow rate capable of generating GLT in human airways. Calculated unidirectional airflow rates equal to or greater than 1 m/s are predicted to be achieved in generation 0–6 airways during expiration and generations 0–4 during inspiration (Table 1). However, a net expiratory versus inspiratory velocity difference of greater than 1 m/s was not calculated over the respiratory cycle at rest. Note that the calculations of net respiratory cycle airflow rate favoring expiratory flow did not factor in the duration of expiratory versus inspiratory cycles, predicted to be shorter for expiration. Therefore, it is not clear whether normal human subjects generate significant net GLT favoring mucus clearance at rest. However, our calculations for net airflow rates during exercise demonstrate that a > 1 m/s expiratory versus inspiratory net flow rate difference is achieved down to the tenth generation airway. Thus, GLT may be significant during exercise in humans.

Our in vitro system also permitted studies of a key variable that affects both cilia- and cough-mediated mucus clearance with respect to GLT, i.e., mucus concentration. Note, hyperconcentration of mucus impairs MCC and CC and plays an important role in the pathogenesis of muco-obstructive lung diseases (30). Studies of the effect of mucus concentration on GLT efficiency demonstrated that hyperconcentrated mucus exhibited significantly slower transport velocities during airflow pulses and a larger recoil upon cessation of airflow pulses, producing a significantly decreased net displacement (transport) per air pulse (Figure 3).

The reduction in GLT-mediated transport as mucus becomes hyperconcentrated is congruent with previous biophysical studies of mucus properties as a function of mucus concentration. First, elastic mucus is essential for normal GLT, as elasticity maintains the mucus layer structure and mediates wave motion during airflow. Conversely, purely viscous fluids thin rapidly, dissipate wave motion, and exhibit slower transport with airflow-induced shear (10, 51). Rheological studies have shown that the elastic modulus of mucus increases exponentially with increased mucus concentrations (52). We speculate that hyperconcentrated mucus (90 mg/mL) exhibited dramatically slower GLT in part because mucus elasticity reached levels that resisted shear deformation during airflow. Our data demonstrating hyperconcentration-dependent reductions in GLT efficiency support observations made by Kim and colleagues that mucus simulants with higher elasticity were also transported more slowly at all airflow velocities (10). Second, when mucus concentration increases, osmotic compression of cilia and increased mucus–cell surface adhesive interactions occur, likely dominating GLT rates at high mucus concentrations (30, 31). Thus, the optimal mechanism for clearance of hyperconcentrated mucus is cough, utilizing the very high shear forces generated to strip adherent mucus off proximal airway surfaces.

While our unidirectional in vitro GLT studies advance our understanding of transport by unidirectional velocity airflow, there are limitations to our experimental design, including (a) a lack of bidirectional airflow (inhalation and exhalation) needed to measure net GLT and (b) airway surface geometry (i.e., flat vs. curved surfaces). To address these limitations, in vivo animal studies were used to investigate the relative roles of GLT versus MCC. The first model to test this notion employed SO_2_ inhalation, a maneuver that produces an airway epithelium devoid of lumen-facing ciliated cells in mice (41, 53) (Figure 5C). Three days after SO_2_ exposure, spontaneously breathing SO_2_-exposed mice exhibited mucus clearance rates approximately 50% of those in sham-treated control mice. Importantly, mucus clearance in SO_2_-exposed mice studied in the absence of breathing, i.e., immediately following death, was essentially abolished. These data demonstrate that the mucus transport measured in SO_2_-treated mice during breathing reflected mucus flow mediated by GLT.

These findings were confirmed in a genetic mouse model of ciliary immobility, i.e., a PCD mouse model. As in the SO_2_ mouse model, live breathing PCD mice exhibited significant basal mucus clearance — approximately 80% of the clearance rate of control mice during breathing conditions. Again, measurements of mucus clearance during nonbreathing conditions (i.e., after euthanasia) failed to detect mucus clearance in PCD mice, whereas rates were preserved in non-PCD control, nonbreathing mice. Of note, the Dnaic1 PCD mice do not exhibit spontaneous lung disease, and we hypothesize that GLT contributes to the absence of a lung phenotype in these mice (44).

Finally, at birth, mouse airways exhibit significantly reduced ciliation with cilia covering approximately 20% of the surface area (23). The reduction in ciliated cell numbers is compounded by poor coordination of cilia beating and disorganized mucus flow. Our data in breathing versus nonbreathing neonatal mice demonstrate that GLT plays a pivotal role in compensating for defects in cilia maturation in the neonatal period. Similar conclusions emerged from data from neonatal ferrets.

The mouse data conclusively demonstrate GLT under physiologic conditions in vivo and emphasize the importance of GLT under tidal breathing conditions in mice with defective ciliary activity. To place the mouse in vivo flow rates into perspective with respect to our calculated human respiratory cycle flow rates (Table 1), we calculated flow rates through mouse tracheae (1 mm diameter) from previous plethysmography studies (54). These calculations predict a maximum expiratory flow rate of approximately 2 m/s and an inspiratory flow rate of approximately 0.5 m/s. Thus, without factoring in small differences in expiratory versus inspiratory cycle intervals, the shape of the expiratory or inspiratory flow rate profiles, or mouse airway mucus concentrations, the in vivo mouse data from PCD mice suggest that a net respiratory cycle flow rate advantage favoring expiration of ≥ 1.5 m/s provides robust GLT at rest.

The data describing the importance of GLT in mucus transport in mouse models with absent/defective cilia have implications for the pathophysiology of people with PCD (pwPCD). Our data demonstrating GLT-mediated mucus clearance in the absence of ciliary beating in these mouse models support the notion that, in pwPCD, residual lung mucus clearance might depend on both GLT and CC (33, 55). Reports of basal mucus clearance rates in PCD subjects over a span of ages in vivo vary, ranging from complete absence of mucus transport to a fraction of normal human MCC rates (56, 57). Measurements of GLT in pwPCD as a function of PCD genotype, mucus concentration, and exercise regimen may explain some of the variability reflected in previous reports.

Our data also have implications for strategies to improve mucus clearance in diseases associated with cilial loss or dysfunction. First, postural drainage is used to clear thickened mucus from the lungs of patients with muco-obstructive lung disease (15, 58–60), including PCD (58). Our studies demonstrated that cilia-mediated clearance is largely independent of body orientation. However, GLT clearance was heavily influenced by gravity, e.g., GLT was impaired in the heads-up position and accelerated in the heads-down position. These data provide a rationale for the role of body position in physiotherapy (61). Second, strong positive correlations were observed between net expiratory airflow velocities and respiratory rates versus the magnitude of GLT-mediated mucus clearance in a large fraction of airways in the lung (generations 0–11). These data underscore the contribution of GLT to the effectiveness of exercise as a component of physiotherapy (62, 63).

We also identified therapeutic strategies to improve GLT. Restoring airway mucus concentrations to normal levels is the simplest approach to achieve a significant increase in the efficiency of GLT. Hydrating the airway surfaces using inhaled osmotic agents, such as hypertonic saline (64, 65) or mannitol (66), and/or agents that inhibit sodium (and fluid) absorption (67) are candidates. Our data also suggest that muco-active agents that decrease mucin viscoelasticity (reducing agents) and mucus–surface interfacial forces (surfactants) (36) may improve GLT associated with modest mucus dehydration/hyperconcentration. These findings support the therapeutic potential of both hydrating and mucin-modifying agents in muco-obstructive diseases like CF and PCD.

Our study has several limitations. First, the respiratory flow rates for each airway generation (Table 1) were calculated from Weibel’s static model of the human airway (at total lung capacity [TLC]), which limits the ability to calculate time-varying flow rates during inhalation versus exhalation. Second, our in vitro studies were performed under conditions of laminar airflow (i.e., Reynolds number < 2,000), whereas higher airflow velocities in the larger, proximal airways (Table 1) likely produced transitional or turbulent flow regimes during exercise. However, this missing feature of our modeling may underestimate the efficacy of GLT in proximal airway regions, as turbulence introduces chaotic eddies and increased shear stress at the gas–liquid interface, potentially amplifying mucus wave formation and propulsion in a 2-phase flow system (16, 51). Third, the in vitro airflow studies were performed at mucus concentrations (10 mg/mL) representative of large airways (30). As data become available for mucus concentrations in the more distal airways, future studies need to investigate GLT rates utilizing peripheral airway mucus concentration, predicted to be more dilute than 10 mg/mL (68). Fourth, the in vitro model used here (poorly differentiated HBE cultures with uncoordinated ciliary beating, akin to the situation in PCD) does not replicate physiologically coordinated ciliary activity. Accordingly, the relative contribution of GLT compared with ciliary beating to mucus clearance in these in vitro studies could not be quantified in this system. Finally, in addition to cilia loss, the SO_2_-exposed mouse protocol used in this study may generate inflammatory responses. However, the replication of our findings of similar velocities of GLT-mediated mucus flow in the PCD mouse (i.e., without SO_2_) mitigates this concern.

In summary, our data support a third mode of mucus clearance in the mammalian lung, i.e., GLT. Whereas GLT likely contributes minimally to mucus clearance rates in the normal lung with intact ciliary beating, GLT may emerge as the predominant basal mucus transport mechanism in pwPCD and acquired ciliary dysfunction (e.g., smoking or infection), with its efficacy further magnified by exercise (69, 70). An understanding of GLT may suggest optimization of current physical therapy approaches to improve the health of individuals with dysfunctional cilia — specifically, postural drainage and exercise. Finally, maneuvers to hydrate and/or reduce the viscoelasticity of mucus in individuals with PCD may improve their respiratory health.

## Methods

### Sex as a biological factor.

While sex was not considered a biological variable, both male and female animals were used in all experiments.

### Cell culture.

Primary HBE cells for these studies were provided by the University of North Carolina (UNC) Marsico Lung Tissue Core Facility under the auspices of the UNC IRB. Deidentified cells from both males and females were plated on the basolateral side of commercially available supports (Snapwell, Costar 3407) at a density of approximately 300,000 cells per/cm^2^. Cells were propagated, differentiated, and maintained using previously published protocols (71). Cultures were maintained at 37°C, 5% CO_2_, and >95% humidity using a well-characterized airway differentiation media (UNC Air Liquid Interface from UNC’s Marsico Lung Institute Tissue Core; https://www.med.unc.edu/marsicolunginstitute/wp-content/uploads/sites/547/2020/11/Primary-HBE-Instructions-June-2020.pdf), replaced 3 times per week for 4 weeks. To isolate GLT-mediated mucus transport in HBE cell cultures, we addressed the confounding effect of cilia-mediated mucus movement. In our circular culture inserts, well-differentiated HBE cultures exhibit coordinated ciliary beating, resulting in circular mucus flow patterns, often referred to as “mucus hurricanes,” which prevented reliable quantification of unidirectional cilia-mediated transport. Therefore, in this study, we prescreened and utilized poorly differentiated HBE cultures that display slow and random ciliary movement, producing negligible net unidirectional displacement, observed using particle tracking approaches previously described (72). For studies utilizing CF bronchial cultures, cells were harvested, cultured, and studied under similar conditions.

### GLT imaging chamber and system design.

The chamber that housed the airway cultures during the GLT experiment (Figure 1, B–D) was constructed from polydimethylsiloxane and polycarbonate to facilitate a variety of culture supports and airflow channel geometries. For the studies described here, the airflow channel was 0.7 mm high and 10 mm wide. This system was designed to subject the luminal airway epithelial surface to a variety of airflow velocities. In these studies, unidirectional flow across the cell surface was produced using the negative pressure (<50 mTorr) generated by a controlled high-volume vacuum pump (model 117, Labconco). This configuration facilitated laminar flow across the epithelium surface (Figure 1D). The bottom of the chamber contained a 20 × 40 × 0.1 mm glass slide used for visualization. A butyl rubber gasket, laser cut to the size of the chamber, was positioned between the chamber and the microscope stage to ensure the chamber was airtight. The completed chamber was clamped to a custom support affixed to the stage of an inverted epifluorescence microscope (Nikon Ti-Eclipse) (Figure 1C). The flow rate during the experiment was regulated by a digital, software-controlled, needle flow valve (SCPV-1-3, Clippard), which could vary the flow from 0 to 50 m/s. A high-speed solenoid was connected between the needle valve and the vacuum pump and was used to control the start and end of the cough pulse. The resulting flow rate and air volume during the air pulse were measured in real time using an in-line mass flow meter (Zephyr, Honeywell). To prevent mucus and culture dehydration, the air at the input of the chamber was humidified to > 95% using a high-volume, noncondensing, humidifier (Levoit). During the GLT pulse, the solenoid opened for a predetermined duration between 1 and 5 s during the air pulse sequence. Labview software and data acquisition hardware (National Instruments) were used to program the digital needle valve positions, record the flow rate data from the mass sensor, and control the timing between the microscope software (Nikon Elements), camera (Zyla 4.2 sCMOS, Andor), and the solenoid-controlled pump. While the entire microscope and air pulse control system was located on an antivibration table, the vacuum pump used to generate the negative pressure air pulse was positioned on an adjacent table to reduce vibrations during the imaging studies.

### Mucus conditions.

In these studies, we exploited the observation that well-differentiated human airway cultures produce an endogenous mucus layer that does not clear and becomes dehydrated over time (30, 31). This allowed us to study mucus at specific concentrations from normal (10 mg/mL organic solids) to CF-like levels (90 mg/mL organic solids). In these studies, we washed the apical surface of the culture (PBS, 3 times, for 15 min) and allowed cultures to achieve the desired mucus concentration before the study. In our experience, mucus concentration in cultures reaches 10 mg/mL in approximately 2 days, 40 mg/mL in approximately 5 days, and 90 mg/mL in approximately 10 days following washing. After washing, the mucus concentration of each donor preparation was routinely measured until the desired concentration was reached. To determine the in vitro mucus concentration during this concentration protocol, we sampled the mucus of parallel cultures using previously published methods (36). Briefly, sterile, preweighted, cellulose mucus-binding mesh (Kimberly Clark) was placed on a replicate culture the night before the study and allowed to incubate overnight. This mesh was peeled off the surface and weighed before and after drying, and mucus concentration was then calculated as the dry mass minus the wet mass (31).

### In vitro GLT studies.

For mucus tracking, 100 nL of fluorescent polystyrene particles (Invitrogen) in PBS was embedded in the mucus layer using an ultrasonic nebulizer, as previously described (64). The embedded fluorescent particles in mucus were tracked using high-speed video microscopy (Zyla 4.2 sCMOS, Andor; ×10 objective, Nikon Ti-Eclipse) at 100 fps. At the start of the pulse, the computer-controlled solenoid was opened, allowing the flow of humidified (>90% relative humidity) room air (~25°C) to flow across the culture surface at the desired velocity and pulse length. At the end of the air pulse, the solenoid was closed, and any recovery of the mucus movement (i.e., elastic recoil) was recorded over the following 5 s. Particle trajectories were analyzed with Trackmate (ImageJ) to quantify mucus displacement, and baseline ciliary movement in poorly differentiated cultures was subtracted using custom Matlab software. The Matlab algorithm calculated vector components (velocity and direction) of ciliary movement in the absence of airflow, averaging displacements over 10–20 particles per field, and subtracted these from total displacement under airflow to isolate GLT-mediated transport (Figure 2), calculated as the net distance from the start of the pulse to the final bead position after the recovery phase. Any bead that could not be tracked for the complete duration of the protocol (prepulse to end of the recovery), including going out of focus or being displaced out of the field of view, was automatically excluded from the analysis.

### Mucus-modulating agents.

Studies investigating the effects of mucus-modifying agents were performed to assess GLT enhancement. Here, either a reducing agent (10 mM DTT) or surfactant (0.01% w/v NP-40) was aerosolized (100 nL) onto the surface of the epithelium using an ultrasonic nebulizer (Aeroneb, Aerogen) 30 min before mounting in the GLT chamber. The effect of these agents on bulk mucus viscoelastic properties was measured using a standard cone-and-plate rheometer (DHR-3, TA Instruments) with isolated mucus at 40 mg/mL.

### Animal studies.

Mice and ferrets used in this study were approved by the UNC IACUC. All breeding, husbandry, and experiments were performed according to NIH guidelines for the care and use of animals in biomedical research. In vivo mucus clearance was assessed in C57BL/6 (Charles River Laboratories) SO_2_-treated and Dnaic1-deficient PCD mice (all 8–12 weeks) and neonatal C57BL/6 mice (P1–P2) and ferrets (P1–P2). Before the study, all animals had free access to both food and water and were on a 12-hour light/12-hour dark sequence. For studies in living, breathing animals, the animals were anesthetized by either xylazine (30 mg/kg) and ketamine (75 mg/kg) administered intraperitoneally or 3.0% isoflurane delivered intranasally using an isoflurane vaporizer (Anesco) outfitted with an in-line humidifier. In studies with nonbreathing animals, the animals were euthanized by an overdose of ketamine/xylazine before radiotracer delivery. All animals (living or after euthanasia) were maintained at a body temperature of 37°C during the experimental procedure. In living animals, breathing frequency was measured using an external physiological monitoring system (model 75-1500, Harvard Apparatus).

### Preparation and delivery of ^99m^TC-SC particles.

TechneScan Sulfur Colloid Kits (CIS-US Inc.) were used to prepare the ^99m^TC-SC particles using ^99m^TC isotopes provided by the UNC Department of Radiology. Small volumes (150 nL) of ^99m^TC-SC were slowly deposited in the distal trachea (at the mainstem) of anesthetized or euthanized animals via a calibrated microcannula made from polyethylene tubing (0.6 mm OD, PE-10, Warner Instruments). Once delivered, animals were placed on the γ camera for image acquisition, typically < 30 s.

### In vivo mucus clearance in animals.

Mucus clearance studies were performed using a custom-made γ camera with a customized gantry that allowed imaging when the animal was placed horizontally or vertically (heads-down or heads-up). The detector was based on a standard scintillation camera design using pinhole collimation (73). The crystal was composed of an array of 1.6 mm × 1.6 mm × 8 mm elements, which were fused using a reflective epoxy (Saint Gobain). Output from the scintillation crystal was coupled to a 5 inch position-sensitive photomultiplier (PMSPMT; model R2392, Hamamatsu) with a useful field of view of 10.4 cm and intrinsic resolution of 1.8 mm for 140 keV photons. The detector was fitted with a custom-designed pinhole collimator with a 10 cm focal length using a 0.5 mm diameter tungsten aperture. The entire γ camera was mounted on a computer-controlled 360° mechanical stage (Newport), allowing us to study mucus clearance against gravity. The signals from the detector were acquired using a data acquisition board (National Instruments) and processed using KMAX software (Sparrow).

Animals were positioned on a magnetic frame for rapid and precise mounting to the γ camera. Each animal was positioned 2 cm from the camera and provided an image field of view of approximately 2.1 cm, which covered the area approximately 5 mm below the bifurcation of the main branches to the top of the trachea. This was arranged to eliminate the interference of radiotracer that would deposit in the stomach as it was cleared from the airways. The resolution with this system, using a 0.5 mm pinhole aperture, was 0.32 mm. Typical depositions of ^99m^TC-SC were 150 nL in volume containing approximately 10 μCi of activity. On average, this provided each acquisition with approximately 3,000 counts within the photopeak. Subcutaneous ^99m^TC fiducial markers (see Figure 5A), placed prior to radiotracer airway deposition, were imaged simultaneously along with the ^99m^TC-SCs and served to coregister images in case of animal movement during the experiments.

### Tracheal bead clearance.

Tracheal mucus clearance in neonatal mice and ferrets was measured using a previously described method (45). Briefly, 3 μm fluorescent particles (Molecular Probes FluoSpheres, Nile Red, Invitrogen) were focally deposited in the mainstem bronchi of neonatal mice (P1–P2) or ferrets (P1–P2) that were either anesthetized (breathing) or immediately following euthanasia. A total of 50 nL was deposited in the trachea of mice and 100 nL in ferrets. After 15 min, the entire trachea was removed, and the tissue was dissolved using 3 M KOH. The number of fluorescent particles remaining in the trachea of each animal was counted optically, and percentages were calculated as the difference between the number of particles injected and the number of particles recovered.

### Airway modeling.

The morphology of the human tracheobronchial tree was modeled using Weibel’s model (26), a symmetric dichotomous branching system comprising 24 generations. This model provides average diameters *d(z)* for each generation *z* in millimeters at TLC. To calculate airflow velocity during exhalation, airway diameters were adjusted to reflect changes at different lung volumes, based on bronchographic measurements of bronchial caliber variations during respiration, as described by Marshall and Holden (14). Based on this work, airflow velocities during exhalation were calculated from airway diameters at functional residual capacity, using the reported percentage decreases: 13% for airways < 1.7 mm, 16% for airways 1.7–3.5 mm, 10% for airways 3.5–7 mm, and 7% for airways > 7 mm. For inhalation, the unadjusted TLC diameters from Weibel’s model were used directly.

Airflow velocity *ν(z)* for each generation *z* was computed as *ν(z)* = *Q/A(z)*, where *Q* is the volumetric flow rate at the trachea. Flow rates of 15 L/min (0.00025 m³/s) and 75 L/min (0.00125 m³/s) were used, assuming incompressible flow, constant volume flow across generations, and uniform velocity distribution within each generation. *A(z)* is the cross-sectional airway during either inspiration or expiration.

### Chemicals.

Except where noted, all chemicals were purchased from MilliporeSigma.

### Statistics.

Data were analyzed using GraphPad Prism (v9.0), with 1-way ANOVA for comparing multiple GLT displacements, followed by Tukey’s post hoc test (*P* < 0.05). Student’s unpaired 2-tailed *t* tests were used for pairwise comparisons. All experiments included at least 3 biological replicates, with sample sizes indicated in the figure legends. In all analyses, a *P* value of less than 0.05 was considered statistically significant. The data are presented as the mean ± SD.

### Study approval.

The experimental procedures on animals were approved by the UNC IACUC. Animal experiments were conducted in accordance with the NIH Guide for the Care and Use of Laboratory Animals (National Academies Press, 2011). Airway cells were obtained from nondiseased human donor lungs through the National Disease Research Interchange with permission of exemption from the UNC IRB.

### Data availability.

Matlab code used to process the ImageJ Trackmate files is available at https://github.com/UNCMLI/AirTrack (commit ID d98113a). Values for all data presented in the graphs are available in the Supporting Data Values file.

## Author contributions

BB, RCB, and BRG conceived and planned the study. SKS, MG, IB, TDR, KB, NO, NG, HPG, BY, DAE, BRG, BB, and DSL generated, analyzed, and interpreted the data. SKS, RCB, and BB wrote the manuscript, and all authors reviewed the manuscript.

## Conflict of interest

The authors have declared that no conflict of interest exists.

## Funding support

This work is the result of NIH funding, in whole or in part, and is subject to the NIH Public Access Policy. Through acceptance of this federal funding, the NIH has been given a right to make the work publicly available in PubMed Central.

- NIH R01HL125280.
- NIH P30DK065988.
- NIH P01HL164320.
- Cystic Fibrosis Foundation (Button19G0).
- Cystic Fibrosis Foundation Therapeutics (Button07XX0).

## Supplementary Material

Supporting data values

## Figures and Tables

**Table 1:**
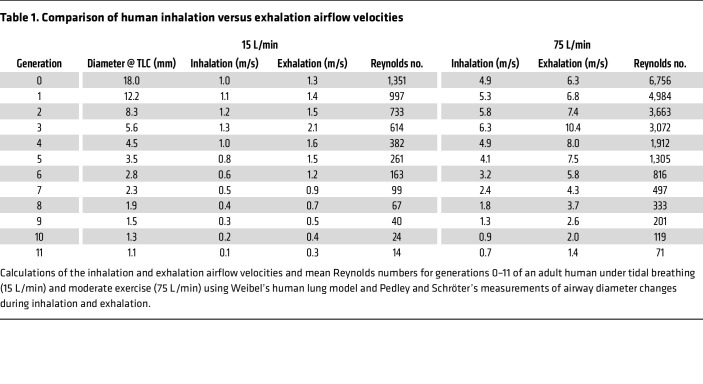
Comparison of human inhalation versus exhalation airflow velocities

## Version 1 03/17/2026

In-Press Preview

## Version 2 05/15/2026

Electronic publication
